# Cellular origin of the viral capsid-like bacterial microcompartments

**DOI:** 10.1186/s13062-017-0197-y

**Published:** 2017-11-13

**Authors:** Mart Krupovic, Eugene V. Koonin

**Affiliations:** 1 Department of Microbiology, Institut Pasteur, 25 rue du Dr. Roux, 75015 Paris, France; 2 National Center for Biotechnology Information, National Library of Medicine, National Institutes of Health, Bethesda, MD 20894 USA

## Abstract

**ᅟ:**

Bacterial microcompartments (BMC) are proteinaceous organelles that structurally resemble viral capsids, but encapsulate enzymes that perform various specialized biochemical reactions in the cell cytoplasm. The BMC are constructed from two major shell proteins, BMC-H and BMC-P, which form the facets and vertices of the icosahedral assembly, and are functionally equivalent to the major and minor capsid proteins of viruses, respectively. This equivalence notwithstanding, neither of the BMC proteins displays structural similarity to known capsid proteins, rendering the origins of the BMC enigmatic. Here, using structural and sequence comparisons, we show that both BMC-H and BMC-P, most likely, were exapted from bona fide cellular proteins, namely, PII signaling protein and OB-fold domain-containing protein, respectively. This finding is in line with the hypothesis that many major viral structural proteins have been recruited from cellular proteomes.

**Reviewers:**

This article was reviewed by Igor Zhulin, Jeremy Selengut and Narayanaswamy Srinivasan. For complete reviews, see the Reviewers’ reports section.

**Electronic supplementary material:**

The online version of this article (10.1186/s13062-017-0197-y) contains supplementary material, which is available to authorized users.

## Findings

**Fig. 1:**
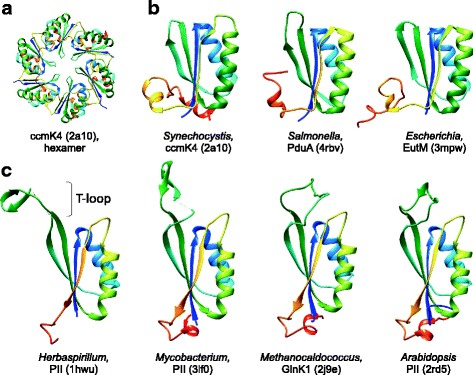
Structural comparison of the major shell protein (BMC-H) of bacterial microcompartments with the PII signaling proteins. **a**. Hexameric assembly of the BMC-H from *Synechocystis* sp. PCC 6803. **b**. BMC-H proteins from carboxysome, Pdu, and Eut microcompartments. **c**. PII signaling proteins from bacteria (*Herbaspirillum* and *Mycobacterium*), archaea (*Methanocaldococcus*) and plants (*Arabidopsis*). All structures are colored using the rainbow scheme from blue (N-terminus) to red (C-terminus) and indicated with the corresponding PDB identifiers

**Fig. 2:**
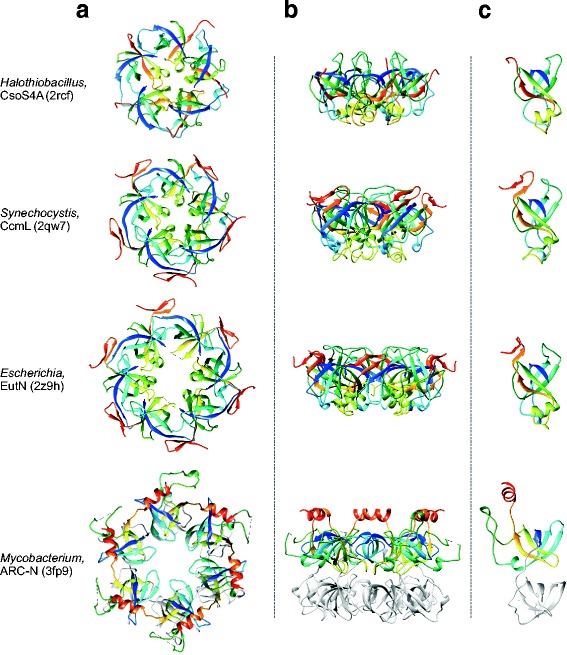
Structural comparison of the minor shell protein (BMC-P) of bacterial microcompartments with the OB-fold domain of the ARC ATPase from *Mycobacterium tuberculosis*. **a**. Structures of the pentameric or hexameric assemblies viewed from the top. **b**. Structures of the pentameric or hexameric assemblies viewed from the side. **c**. Structures of the monomers. All structures are colored using the rainbow scheme from blue (N-terminus) to red (C-terminus) and indicated with the corresponding PDB identifiers. The second OB-fold domain of the ARC ATPase is shown in light grey for convenience

Two classes of icosahedral compartments have been identified in bacteria and archaea that show striking morphological resemblance to viral capsids. The first class includes the encapsulin nanocompartments (24 or 32 nm in diameter) that are structurally similar to and possibly derived from the HK97-like major capsid proteins of tailed bacterial and archaeal viruses of the order *Caudovirales* [1, 2]. The second class consists of much larger, 40–500 nm in diameter, bacterial microcompartments (BMC), which encapsulate enzymes for a variety of metabolic pathways; compartmentalization is thought to optimize the performance of these specialized reactions [3–5]. The three best-studied types of BMC include (i) carboxysomes that package the CO_2_ fixation enzyme ribulose 1,5-bisphosphate carboxylase/oxygenase (RuBisCO), (ii) 1,2-propanediol utilization (Pdu) BMC which optimize the coenzyme B12-dependent catabolism of 1,2-propanediol as a growth substrate, and (iii) the ethanolamine utilization (Eut) BMC responsible for B12-dependent degradation of ethanolamine, which can serve as a sole source of both carbon and nitrogen. Similar to some complex viral capsids, such as those of adenoviruses [6, 7], tectiviruses [8] or virophages [9], the BMC are constructed from two non-homologous, structurally unrelated shell proteins. Homohexamers of the major BMC protein, BMC-H, form hexagonal capsomers (Fig. 1a), whereas pentamers of the minor BMC protein, BMC-P, form pentagonal capsomers (Fig. 2a) [10]. These two BMC proteins correspond, respectively, to the major and minor capsid proteins of viruses with icosahedral capsids [11, 12]. Furthermore, some BMC contain BMC-T proteins, dimeric derivatives, in which two BMC-H domains are fused within a single polypeptide, so that a hexagonal assembly is formed by the BMC-T trimer, rather than a hexamer of BMC-H [13]. The presumed icosahedral symmetry of the BMC has been recently confirmed by the crystal structure of an intact shell from *Haliangium ochraceum*, showing that the hexagonal BMC-H and BMC-T capsomers form the facets, whereas the pentagonal BMC-P capsomers occupy the 12 vertices of a *T* = 9 icosahedron [14]. The BMC-H and BMC-P proteins are homologous across all known types of BMC [3–5, 14]. However, no similarity to any known viral capsid protein has been observed thus far, and the origin of these remarkable cellular structures remains enigmatic.

To gain insights into the potential origin of the BMC, we performed structural comparisons of the BMC-H and BMC-P proteins to available protein structures using the DALI server [15]. As expected, corresponding proteins from different BMC types were found as the closest homologs (Figs. 1b and 2), and neither of the two proteins displayed significant structural similarity to known viral capsid proteins. Additionally, however, searches initiated with the carboxysomal, Pdu or Eut BMC-H proteins resulted in multiple, highly significant matches to the family of PII signal transduction proteins (Fig. 1c) that are responsible for the regulation of nitrogen metabolism in archaea, bacteria and plants [16]. For instance, when the structure of the BMC-H protein from *Halothiobacillus neapolitanus* (PDB id: 2G13) was used as a query, PII proteins from *Herbaspirillum seropedicae* (PDB id: 1HWU), *Mycobacterium tuberculosis* (PDB id: 3LF0) and *Anabaena variabilis* (PDB id: 3DFE) were retrieved as the best hits (besides other BMC-H proteins) with the Z score of 7.9, 7.7 and 7.7, respectively, despite low sequence similarity (11–17% identity; Additional file 1). The major structural difference between the PII and BMC-H proteins is the absence of the ATP/ADP-binding T-loop [17] in the latter (Fig. 1). Notably, PII proteins function as homotrimers [18], indicating that the tendency to form higher-order oligomers is common to both BMC-H and PII proteins. Considering that BMC proteins have been detected in only ~17% of known bacteria and none in archaea [19], whereas PII proteins are among the most ancient, ubiquitous and versatile components of signaling systems in nature [17], it appears most likely that BMC-H proteins evolved from PII-like proteins. However, it cannot be ruled out that the two families of cellular proteins have diverged from a common ancestor in a more distant past.

Searches against the DALI database initiated with the BMC-P proteins resulted in multiple significant hits to various bacterial proteins with the oligonucleotide/oligosaccharide-binding (OB) fold (Fig. 2), a five-stranded closed β-barrel, one of the most ancient and widespread structural folds found in a wide range of functionally diverse proteins [20, 21]. The BMC-P proteins showed the highest structural similarity to translation initiation factor IF-1 (PDB id: 4QL5; Z score 6.4) and the N-terminal substrate recognition domains of proteasomal ARC ATPases from actinobacteria (PDB id: 2WFW; Z score 6.1). The relationship between BMC-P and OB-fold proteins could be also established using sensitive profile-profile searches with HHpred [22]. For instance, a search initiated with the carboxysomal BMC-P protein (WP_012823797) resulted in multiple matches, as the best hits (besides other BMC-P proteins), to the sequence profiles of OB fold domains from various proteins, including HupF/HypC-like proteins (SCOPe profile: d3vyta_), phenylalanyl-tRNA synthetase (SCOPe profile: d1b70b3), and tetratricopeptide repeat protein 5 (TTC5; Pfam accession number: PF16669.4), albeit with moderately significant probabilities of 66–76% (Additional file 1). Conversely, when the search was initiated with the sequence of the OB fold domain of TTC5, the SCOPe profile (d2rcfe_) of carboxysomal BMC-P protein was retrieved with the probability of 70.5%. Although BMC-P normally form flat pentagonal assemblies (Fig. 2a), the BMC-P protein from Eut BMC was crystalized as a pseudohexagonal hexamer (PDB id: 2Z9H; Fig. 2) [23], although it is a pentamer in solution [24]. Remarkably, the substrate recognition domain of the ARC ATPases consists of two tandem OB-fold domains [25, 26], both of which can be superimposed with BMC-P, and forms hexameric rings resembling those formed by EutN (Fig. 2).

The above observations strongly suggest that both BMC proteins, BMC-H and BMC-P, have been exapted from bona fide cellular proteins, namely, PII signaling and OB-fold domain-containing proteins, respectively, to function as structural components of the BMC. Notably, genes encoding both PII proteins and OB fold-containing ARC ATPases co-occur in some bacterial genomes, e.g. *Mycobacterium tuberculosis* (Figs. 1 and 2), which would provide an opportunity for their co-evolution and concerted refunctionalization. The case of BMC is a striking example of de novo evolution of complex, icosahedral virus-like particles from cellular proteins which is consistent with our previous conclusion that many if not most viral capsid proteins were derived throughout the course of evolution from diverse cellular proteins [1].

## Methods

The protein structures were downloaded from the Research Collaboration for Structural Bioinformatics (RCSB) Protein Data Bank (PDB) (www.rcsb.org). Structure-based searches were performed using the DALI server [15]. Structural similarities between proteins were evaluated based on the DALI Z score, which is a measure of the quality of the structural alignment. Z scores above 2, i.e., two SDs above expected, are usually considered significant [27]. The relevance of the matches was evaluated further by visual inspection of structural alignments. Structures were aligned using the MatchMaker algorithm implemented in the University of California, San Francisco (UCSF) Chimera package [28] and were visualized using the same software. Profile-against-profile searches were performed using HHpred [22] against different protein databases, including Pfam, PDB, CDD, and SCOPe, which are available via the HHpred website.

## Reviewer’s reports

### Reviewer 1: Igor Zhulin, University of Tennessee

In this paper, Krupovic and Koonin report that major shell proteins that form bacterial microcompartments (BMC) are structurally similar to ubiquitous cellular proteins, such as PII and IF-1, and in some cases can be linked to those by sequence similarity as well. They propose that BMC-H and BMC-P proteins originated from cellular proteins with ancient folds - ferredoxin-like (PII) and OB. This is an interesting observation and supporting evidence (structural similarity revealed by DALI and sequence similarity revealed by HHpred) is fairly convincing.

RESPONSE: *We appreciate the positive assessment of our work and thank Dr. Zhulin for constructive comments.*

1. Line 52: Should be “domains”

RESPONSE: *Corrected.*

2. Lines 65, 68, 79, 86, 89: it seems that “initiated” and “query” would be better word choices than “seeded” and “seed”, although it is a matter of taste, I suppose.

RESPONSE: *Changed.*

3. Line 71: should be “6–11% identity” unless something else was meant.

RESPONSE: *Corrected.*

4. Lines 73–76: Perhaps, it would be worthwhile mentioning that PII proteins function as homotrimers (Llacer et al., PNAS 2007, 104: 17,644). Thus, tendency to higher-order oligomerization is a feature common to both BMC-H and PII.

RESPONSE: *Thank you for pointing this out. This is now mentioned in the text and the corresponding reference is cited.*

5. Line 88: indicate that PF16669.4 is a Pfam accession number.

RESPONSE: *Indicated, as suggested.*

6. Lines 89 and 119: should be “SCOPe”.

RESPONSE: *Corrected.*

7. Line 103. This observation is “in line with” the authors’ previous conclusion (Reference 1), but “supports” seems to be an overstatement in the absence of a hardcore evidence directly linking BMC and viral capsid proteins (other than a unique, complex geometrical shape of protein complexes). The authors do use the expression “in line with” in the abstract (line 32) and I suggest using the same wording here.

RESPONSE: *Toned down, as suggested; it is now “consistent with”.*

8. Line 119: should be “Pfam”.

RESPONSE: *Corrected.*

9. Line 119: delete the second instance of the word “databases”.

RESPONSE: *Deleted.*

### Reviewer 2: Jeremy Selengut, University of Maryland

Krupovic and Koonin, in their previously published paper (PNAS, 2017) detailed cases of viral capsid protein evolution from cellular components. Here they address the origins of the structurally capsid-like bacterial microcompartments (BMCs), and find that the two component protein families (BMC-H and BMC-P) also can be traced to other distantly related cellular components with very different functional roles, namely the PII and the OB-fold proteins. The authors provide evidence for these claims from two structural comparison tools: the DALI server and HHpred as well as providing ribbon models for visual comparison. Also noted is the lack of any significant similarity evidence linking these families to families of viral proteins. These observations are straightforward and the conclusions drawn are reasonable given the evidence.

RESPONSE: *We appreciate the positive assessment of our work and thank Dr. Selengut for constructive comments.*

Major:

1. The summary facts of the DALI and HHpred results in the text should be accompanied by figures or tables of the actual output of these programs. Readers should be able to scan these results for themselves and determine that the summary conclusions are valid, seeing the significance scores of the hits that the authors regard as below the significance threshold as well as the ones supporting the author’s claims.

RESPONSE: *In the course of the revision, we repeated the DALI and HHpred searches against the updated databases. The results remained the same, although the score values slightly changed as could have been expected. In the revised manuscript, we included a new Additional file* *1* *, which lists top-30 results of both DALI and HHpred searches using BMC-H and BMC-P proteins.*

(2) Similarly, on line 71, some Z-scores are presented. The authors should put those scores in a broader context detailing why those numbers are regarded as significant, and how they compare to the next-best, but not regarded as significant Z-scores.

RESPONSE: *In the revised manuscript, we added a pointer to Additional file* *1* *, which shows the scores in a broader context. Generally, the Z-scores are highly significant, as scores above 2 are usually considered significant, as mentioned in the Methods section. For BMC-H and BMC-P proteins, diverse PII and OB-fold proteins were retrieved as the top-scoring hits, respectively, as can now be evaluated from the data provided in Additional file* *1*.

Minor:

1. Also on line 71, the sequence similarity is reported as 611% (!), please correct this number.

RESPONSE: *Corrected.*

(2) The digital quality of Figs. 1 and 2 is not good enough for publication. Please provide higher-quality images.
(3) RESPONSE: *The high-resolution TIFF images were automatically downloaded by the Manuscript submission system. However, the original images could be downloaded by clicking on the weblinks provided at the top-right corner of the corresponding pages. In the revised manuscript, we uploaded pdf images, which should be properly displayed.*

### Reviewer 3: Narayanaswamy Srinivasan, Indian Institute of Science, Bangalore

Authors of this manuscript report a fascinating observation on the analogy between bacterial microcompartments (BMC) which encapsulate enzymes and viral capsids. Though the two components of BMC (BMC-H and BMC-P) are functionally similar to the virus capsid proteins, their tertiary structures are entirely different from that of the viral capsid proteins. Indeed, authors clearly show the tertiary structural similarities between BMC-H and PII-signaling protein and between BMC-P and OB-fold domain containing proteins.

RESPONSE: *We thank Dr. Srinivasan for the positive assessment of our work.*

Authors state that “….both BMC proteins, BMC-H and BMC-P, have been exapted from bona fide cellular proteins, namely, PII signaling and OB-fold domain-containing proteins,….”. Do they mean BMC-H & PII signalling protein and BMC-P & OB-fold domain containing proteins are evolutionarily related? If so, it will be nice to show some support from homology searches using sequences or some other arguments. Similarity in tertiary structures between two unrelated proteins is possible as the number of protein domain folds is limited.

RESPONSE: *Indeed, we conclude that BMC-H proteins have been exapted from PII signalling proteins, whereas BMC-P evolved from OB-fold domain-containing proteins. As mentioned in the text, the relationship between BMC-P and OB-fold proteins is also supported by direct sequence profile-profile comparisons using HHpred. In the revised manuscript, we added Additional file* *1* *, which lists the top 30 results of the HHpred search seed with the sequence of a BMC-P protein. More generally, significant structural similarity between two proteins, as presented in this manuscript and supported by DALI Z-scores, is normally perceived as sufficient evidence of common ancestry. Although we certainly agree that the number of protein folds is limited, we are not aware of any convincing demonstration examples where two complex folds would evolve independently from scratch.*

## Additional file

Additional file 1: Results of the DALI and HHpred searches initiated with the structures of the BMC-H (A) and BMC-P (B) proteins or with the sequence of BMC-P protein (C). (PDF 107 kb)

## Abbreviations

BMC: Bacterial MicroCompartment
CDD: Conserved Domain database
OB: Oligonucleotide/Oligosaccharide-Binding (domain)
PDB: protein data Bank
