# Supplementary material for: Cellular origin of the viral capsid-like bacterial microcompartments
Source: Biol Direct. 2017 Nov 13;12:25. doi: 10.1186/s13062-017-0197-y (PMC5683377; doi:10.1186/s13062-017-0197-y)
Supplement: Additional file 1: — Results of the DALI and HHpred searches initiated with the structures of the BMC-H (A) and BMC-P (B) proteins or with the sequence of BMC-P protein (C). (PDF 107 kb) [file 13062_2017_197_MOESM1_ESM.pdf]

A. Top-30 results of the DALI search seeded with BMP-H protein (PDB id: 2a10)

No:	Chain	Z	rmsd	lali	nres	%id	Description
**1:**	**2g13-A**	**24.1**	**0.0**	**95**	**95**	**100**	**MAJOR CARBOXYSOME SHELL PROTEIN 1A;**
**2:**	**5d6v-A**	**18.7**	**0.7**	**89**	**90**	**57**	**CARBOXYSOME SHELL PROTEIN;**
**3:**	**4axj-B**	**17.7**	**0.8**	**86**	**89**	**67**	**ETHANOLAMINE CARBOXYSOME STRUCTURAL PROTEIN;**
**4:**	**4qiv-A**	**17.7**	**0.8**	**86**	**86**	**51**	**BACTERIAL MICROCOMPARTMENTS FAMILY PROTEIN;**
**5:**	**5l38-A**	**17.5**	**1.4**	**90**	**91**	**50**	**MSM0272 - RMM MICROCOMPARTMENT SHELL PROTEIN;**
**6:**	**4liw-A**	**16.9**	**1.2**	**90**	**92**	**48**	**CARBON DIOXIDE-CONCENTRATING MECHANISM PROTEIN CC**
**7:**	**5djb-A**	**16.7**	**0.9**	**87**	**98**	**55**	**MICROCOMPARTMENTS PROTEIN;**
**8:**	**2a1b-A**	**16.2**	**1.4**	**91**	**101**	**52**	**CARBON DIOXIDE CONCENTRATING MECHANISM PROTEIN CC**
**9:**	**3mpw-A**	**16.2**	**1.1**	**88**	**99**	**53**	**ETHANOLAMINE UTILIZATION PROTEIN EUTM;**
**10:**	**3sss-E**	**15.8**	**1.2**	**89**	**106**	**49**	**CARBON DIOXIDE CONCENTRATING MECHANISM PROTEIN;**
**11:**	**4ox6-A**	**15.8**	**1.2**	**89**	**103**	**35**	**CARBON DIOXIDE CONCENTRATING MECHANISM PROTEIN CC**
**12:**	**4rbv-B**	**15.6**	**2.7**	**91**	**94**	**57**	**PROPANEDIOL UTILIZATION PROTEIN PDUA;**
**13:**	**2a10-C**	**15.2**	**1.2**	**89**	**107**	**36**	**CARBON DIOXIDE CONCENTRATING MECHANISM PROTEIN CC**
**14:**	**3vcd-A**	**14.7**	**1.8**	**90**	**184**	**29**	**PROPANEDIOL UTILIZATION POLYHEDRAL BODY PROTEIN P**
**15:**	**3pac-A**	**14.7**	**1.8**	**90**	**183**	**30**	**SHELL PROTEIN;**
**16:**	**3nwg-A**	**13.3**	**2.1**	**83**	**180**	**31**	**MICROCOMPARTMENTS PROTEIN;**
**17:**	**5dih-B**	**11.7**	**1.5**	**88**	**202**	**23**	**MICROCOMPARTMENTS PROTEIN;**
**18:**	**5hpn-A**	**10.3**	**1.6**	**68**	**87**	**57**	**PERMUTED PDUA;**
**19:**	**3i82-A**	**8.3**	**1.6**	**65**	**216**	**23**	**ETHANOLAMINE UTILIZATION PROTEIN EUTL;**
**20:**	**1hwu-B**	**7.8**	**2.6**	**75**	**101**	**16**	**PII PROTEIN;**
21:	2pfd-A	7.8	2.8	86	540	9	FORMIMIDOYLTRANSFERASE-CYCLODEAMINASE;
**22:**	**3dfe-B**	**7.7**	**2.8**	**77**	**83**	**14**	**PUTATIVE PII-LIKE SIGNALING PROTEIN;**
**23:**	**3lf0-B**	**7.7**	**3.3**	**79**	**112**	**13**	**NITROGEN REGULATORY PROTEIN P-II;**
**24:**	**3u27-D**	**7.5**	**1.7**	**69**	**218**	**22**	**MICROCOMPARTMENTS PROTEIN;**
**25:**	**3io0-A**	**7.5**	**2.2**	**73**	**229**	**14**	**ETUB PROTEIN;**
**26:**	**4edi-C**	**7.3**	**1.7**	**69**	**225**	**29**	**ETHANOLAMINE UTILIZATION PROTEIN;**
**27:**	**4i61-A**	**7.3**	**1.6**	**66**	**228**	**23**	**PROPANEDIOL UTILIZATION PROTEIN PDUB;**
**28:**	**2j9e-B**	**7.3**	**3.3**	**77**	**119**	**12**	**HYPOTHETICAL NITROGEN REGULATORY PII-LIKE**
**29:**	**3ta1-D**	**7.2**	**3.3**	**77**	**115**	**17**	**NITROGEN REGULATORY PROTEIN P-II (GLNB-3);**
**30:**	**2xbp-A**	**7.2**	**3.3**	**77**	**113**	**14**	**NITROGEN REGULATORY PROTEIN P-II;**

Hits to BMC-H homologs are shown in Bold; hits to PII-like proteins are in red, whereas the false-positive hit to formimidoyltransferase-cyclodeaminase is shown in plain font. The false-positive hit was evaluated by direct comparison of the protein structures.

B. Top-30 results of the DALI search seeded with BMP-P protein (PDB id: 2rcf)

No:	Chain	Z	rmsd	lali	nres	%id	Description
**1:**	**2rcf-A**	**20.0**	**0.0**	**82**	**82**	**100**	**UNIDENTIFIED CARBOXYSOME POLYPEPTIDE;**
**2:**	**2hd3-B**	**13.1**	**2.3**	**82**	**97**	**39**	**ETHANOLAMINE UTILIZATION PROTEIN EUTN;**
**3:**	**4i7a-D**	**12.4**	**2.4**	**78**	**87**	**32**	**ETHANOLAMINE UTILIZATION PROTEIN EUTN/CARBOXYSOME**
**4:**	**5l37-C**	**12.3**	**2.3**	**81**	**86**	**30**	**MSM0273;**
**5:**	**4jvz-C**	**11.9**	**2.3**	**81**	**97**	**31**	**CARBON DIOXIDE CONCENTRATING MECHANISM PROTEIN;**
**6:**	**4n8x-1**	**11.8**	**2.2**	**81**	**99**	**32**	**CARBON DIOXIDE CONCENTRATING MECHANISM PROTEIN;**
**7:**	**4jw0-B**	**11.8**	**2.3**	**81**	**97**	**36**	**CARBON DIOXIDE CONCENTRATING MECHANISM PROTEIN;**
**8:**	**2qw7-H**	**11.7**	**2.3**	**81**	**98**	**36**	**CARBON DIOXIDE CONCENTRATING MECHANISM PROTEIN CC**
**9:**	**4ql5-A**	**6.4**	**2.1**	**53**	**68**	**17**	**TRANSLATION INITIATION FACTOR IF-1;**
**10:**	**2eif-A**	**6.2**	**2.7**	**54**	**133**	**15**	**PROTEIN (EUKARYOTIC TRANSLATION INITIATION FACTOR**
**11:**	**2wfw-B**	**6.1**	**2.6**	**57**	**125**	**16**	**ARC;**
**12:**	**2dgy-A**	**6.1**	**3.2**	**61**	**111**	**15**	**MGC11102 PROTEIN;**
**13:**	**2wg5-A**	**6.0**	**2.0**	**51**	**87**	**22**	**GENERAL CONTROL PROTEIN GCN4, PROTEASOME-ACTIVATI**
**14:**	**3i4o-A**	**6.0**	**2.1**	**53**	**68**	**17**	**TRANSLATION INITIATION FACTOR IF-1;**
**15:**	**1iz6-A**	**5.9**	**2.8**	**55**	**136**	**15**	**INITIATION FACTOR 5A;**
**16:**	**1fjg-Q**	**5.8**	**2.7**	**56**	**104**	**14**	**30S RIBOSOMAL PROTEIN S17;**
**17:**	**2ot2-A**	**5.8**	**1.6**	**51**	**90**	**16**	**HYDROGENASE ISOENZYMES FORMATION PROTEIN HYPC;**
**18:**	**2n8n-A**	**5.8**	**2.3**	**52**	**72**	**21**	**TRANSLATION INITIATION FACTOR IF-1;**
**19:**	**5mp9-J**	**5.8**	**2.0**	**52**	**386**	**10**	**PROTEASOME SUBUNIT ALPHA TYPE-1;**
**20:**	**1u0l-A**	**5.7**	**3.3**	**57**	**278**	**14**	**PROBABLE GTPASE ENGC;**
**21:**	**3m9b-A**	**5.6**	**2.6**	**56**	**186**	**18**	**PROTEASOME-ASSOCIATED ATPASE;**
22:	3iz6-P	5.6	3.9	65	85	2	40S RIBOSOMAL PROTEIN RACK1 (RACK1);
**23:**	**3h43-I**	**5.6**	**2.1**	**54**	**79**	**17**	**PROTEASOME-ACTIVATING NUCLEOTIDASE;**
**24:**	**5mp9-I**	**5.6**	**3.0**	**55**	**385**	**18**	**PROTEASOME SUBUNIT ALPHA TYPE-1;**
**25:**	**5kzf-H**	**5.5**	**2.6**	**55**	**478**	**18**	**PROTEASOME-ASSOCIATED ATPASE;**
**26:**	**4bpe-0**	**5.5**	**2.4**	**56**	**99**	**23**	**TRANSLATION INITIATION FACTOR EIF-1A FAMILY PROTE**
**27:**	**5mmj-q**	**5.4**	**3.1**	**60**	**86**	**13**	**50S RIBOSOMAL PROTEIN L31;**
**28:**	**2z1c-B**	**5.4**	**1.8**	**51**	**74**	**22**	**HYDROGENASE EXPRESSION/FORMATION PROTEIN HYPC;**
**29:**	**1bkb-A**	**5.3**	**2.7**	**53**	**136**	**11**	**TRANSLATION INITIATION FACTOR 5A;**
30:	2z6b-A	5.3	3.7	62	573	10	TAIL-ASSOCIATED LYSOZYME;

Hits to BMC-P homologs are shown in Bold; hits to OB-fold proteins are in red, whereas the two false-positives are shown in plain font. The false-positives were evaluated by direct comparison of the protein structures. In both cases, the proteins have highly enriched in beta-strands, but do not share the same fold as BMC-P.

C. Top-30 results of the HHpred search seeded with BMP-P protein sequence (WP_012823797)

Query WP_012823797.1 carboxysome peptide A [Halothiobacillus neapolitanus]
Match_columns 83
Searched_HMMs 94372
Date Wed Nov 1 08:13:30 2017

No	Hit	Prob	E-value	P-value	Score	SS	Cols	Query HMM	Template HMM	
**1**	**2RCF_A Crystal Structure of Or**	**100.0**	**4E-35**	**4.3E-40**	**190.9**	**12.7**	**83**	**1-83**	**1-83**	**(91)**
**2**	**2RCF_D Crystal Structure of Or**	**100.0**	**4E-35**	**4.3E-40**	**190.9**	**12.7**	**83**	**1-83**	**1-83**	**(91)**
**3**	**d2rcfe_ b.40.15.1 (E:) Carboxy**	**100.0**	**6E-35**	**6.3E-40**	**186.0**	**12.5**	**80**	**1-80**	**1-80**	**(80)**
**4**	**d4jvzb_ b.40.15.1 (B:) Carbon**	**100.0**	**1E-34**	**1.1E-39**	**189.7**	**12.4**	**83**	**1-83**	**1-88**	**(94)**
**5**	**d2qw7c_ b.40.15.1 (C:) Carbon**	**100.0**	**1.1E-34**	**1.2E-39**	**189.9**	**12.3**	**83**	**1-83**	**1-88**	**(95)**
**6**	**4I7A_E Ethanolamine utilizatio**	**100.0**	**1.1E-34**	**1.2E-39**	**190.0**	**12.3**	**83**	**1-83**	**1-88**	**(95)**
**7**	**5L37_C MSM0273; bacterial micr**	**100.0**	**1.5E-34**	**1.6E-39**	**189.5**	**12.6**	**83**	**1-83**	**1-83**	**(95)**
**8**	**5L37_B MSM0273; bacterial micr**	**100.0**	**1.5E-34**	**1.6E-39**	**189.5**	**12.6**	**83**	**1-83**	**1-83**	**(95)**
**9**	**d2hd3a1 b.40.15.1 (A:1-95) Eth**	**100.0**	**1.9E-34**	**2E-39**	**188.7**	**12.3**	**83**	**1-83**	**1-88**	**(95)**
**10**	**5V74_P1 Ethanolamine utilizati**	**100.0**	**1.7E-34**	**1.8E-39**	**189.4**	**12.0**	**83**	**1-83**	**1-88**	**(96)**
**11**	**d4jw0a_ b.40.15.1 (A:) automat**	**100.0**	**2E-34**	**2.1E-39**	**188.6**	**12.2**	**83**	**1-83**	**1-88**	**(95)**
**12**	**4JVZ_C Carbon dioxide concentr**	**100.0**	**2.5E-34**	**2.6E-39**	**189.4**	**12.5**	**83**	**1-83**	**1-88**	**(99)**
**13**	**4JW0_E Carbon dioxide concentr**	**100.0**	**4.1E-34**	**4.3E-39**	**191.1**	**12.2**	**83**	**1-83**	**1-88**	**(108)**
**14**	**2HD3_B Ethanolamine utilizatio**	**100.0**	**4.3E-34**	**4.5E-39**	**189.6**	**12.1**	**83**	**1-83**	**1-88**	**(103)**
**15**	**2HD3_C Ethanolamine utilizatio**	**100.0**	**4.3E-34**	**4.5E-39**	**189.6**	**12.1**	**83**	**1-83**	**1-88**	**(103)**
**16**	**4N8F_B Carbon dioxide concentr**	**100.0**	**5.3E-34**	**5.6E-39**	**191.6**	**12.5**	**83**	**1-83**	**1-88**	**(112)**
**17**	**2QW7_F Carbon dioxide concentr**	**100.0**	**4.8E-34**	**5.1E-39**	**191.6**	**12.3**	**83**	**1-83**	**1-88**	**(111)**
**18**	**2QW7_H Carbon dioxide concentr**	**100.0**	**4.8E-34**	**5.1E-39**	**191.6**	**12.3**	**83**	**1-83**	**1-88**	**(111)**
**19**	**d4n8x2_ b.40.15.1 (2:) Carbon**	**100.0**	**7.8E-34**	**8.2E-39**	**186.1**	**12.1**	**83**	**1-83**	**1-89**	**(96)**
**20**	**4N8X_B Carbon dioxide concentr**	**100.0**	**1.9E-33**	**2E-38**	**188.1**	**12.1**	**83**	**1-83**	**1-89**	**(109)**
**21**	**4N8X_4 Carbon dioxide concentr**	**100.0**	**1.9E-33**	**2E-38**	**188.1**	**12.1**	**83**	**1-83**	**1-89**	**(109)**
**22**	**PF03319.12 ; EutN_CcmL ; Ethan**	**100.0**	**6.3E-33**	**6.7E-38**	**179.7**	**11.9**	**78**	**1-78**	**1-89**	**(89)**
**23**	**d3vyta_ b.40.14.1 (A:) Hydroge**	**76.7**	**9.6**	**0.0001**	**22.8**	**2.9**	**25**	**31-55**	**20-45**	**(52)**
**24**	**d1b70b3 b.40.4.4 (B:39-151) Do**	**76.6**	**40**	**0.00042**	**22.9**	**7.1**	**48**	**6-56**	**8-56**	**(113)**
**25**	**2XVS_A TETRATRICOPEPTIDE REPEA**	**74.4**	**39**	**0.00041**	**24.9**	**5.9**	**46**	**6-54**	**79-130**	**(166)**
**26**	**PF16669.4 ; TTC5_OB ; Tetratri**	**74.2**	**50**	**0.00053**	**22.8**	**6.2**	**44**	**6-52**	**20-69**	**(114)**
**27**	**2Z1C_B Hydrogenase expression/**	**72.2**	**10**	**0.00011**	**24.5**	**2.4**	**21**	**37-57**	**28-49**	**(75)**
**28**	**d2z1ca1 b.40.14.1 (A:2-72) Hyd**	**69.0**	**18**	**0.00019**	**23.1**	**2.9**	**19**	**37-55**	**27-46**	**(71)**
**29**	**PF01455.17 ; HupF_HypC ; HupF/**	**68.8**	**48**	**0.00051**	**20.9**	**4.6**	**40**	**15-55**	**8-48**	**(66)**
**30**	**3PCO_B Phenylalanyl-tRNA synth**	**66.8**	**77**	**0.00081**	**28.4**	**6.9**	**51**	**6-56**	**46-97**	**(795)**

Hits to BMC-P homologs are shown in Bold; hits to OB-fold proteins are in red.
